# Mitochondria in *SARS-CoV-2* infection: Immune interactions and molecular approaches in the Post COVID-19 condition

**DOI:** 10.1590/1678-4685-GMB-2025-0118

**Published:** 2026-08-28

**Authors:** Maria Clara Barros, Felipe Gouvêa de Souza, Helenize Costalat, Catarina Torres Pinho, Karina Carvalho Marques, Matheus Epifane-de-Assunção, Giovanna C. Cavalcante, Luiz Fábio Magno Falcão, Juarez A. S. Quaresma, Ândrea Ribeiro-dos-Santos

**Affiliations:** 1 Universidade Federal do Pará (UFPA), Instituto de Ciências Biológicas, Laboratório de Genética Humana e Médica (LGHM), Programa de Pós-Graduação em Genética e Biologia Molecular (PPGBM), Belém, PA, Brazil.; 2 Universidade do Estado do Pará (UEPA), Centro de Ciências Biológicas e da Saúde, Laboratório de Doenças Infecciosas e Cardiopulmonares, Programa de Pós-Graduação em Biologia Parasitária na Amazônia, Programa de Long COVID Longa, Belém, PA, Brazil.; 3 Universidade de São Paulo (USP), Instituto de Química, Laboratório de Metabolismo Energético, São Paulo, SP, Brazil.

**Keywords:** Post COVID-19 condition, mitochondria, immune response

## Abstract

Mutations in mitochondrial genes can disrupt key cellular functions and contribute to the development of various complex diseases. The pandemic of COVID-19, caused by the *SARS-CoV-2* infection, has been associated as the cause of certain mitochondrial dysfunctions, including physiological and genetic due to infection processes as increased release of reactive oxygen species (ROS), formation of the NLR family pyrin domain containing 3 (NLRP3) inflammasome, mitophagy impairment and mitochondrial apoptotic pathway. This review compiled the main interactions between *SARS-CoV-2* infection and mitochondria, highlighting mainly the genetic and immunological mechanisms that contribute to the progression of the disease and how the persistence of this inflammatory and dysfunctional state can lead to cardiac, muscular and neurological sequelae, characterizing the post COVID-19 condition.

## Introduction

Derived from millions of years of endosymbiotic evolution and, therefore, holding a central role in eukaryotic catabolic and anabolic metabolism, mitochondria are essential for cellular homeostasis (Suomalainen and Nunnari, 2024). These organelles have a double membrane and their own genome, which 37 genes synthesize the central subunits of four electron transport chain complexes, 22 transfer ribosomal ribonucleic acid (tRNAs), and two ribosomal ribonucleic acid (rRNAs) (Andrews et al., 1999). Additionally, mitochondria may exhibit diverse spatial conformations depending on the metabolic demands of the tissue, ranging from discrete, isolated structures to intricate networks of interconnected organelles (Jenkins et al., 2024). 

Disturbances in their metabolic processes tend to result in serious adverse outcomes, especially when these occur in tissues with high energy demand sustained mainly by oxidative phosphorylation (OXPHOS), such as skeletal muscle, cardiovascular and nervous tissue (Singh 2021; Swalsingh et al., 2022). For instance, diseases such as Amyotrophic Lateral Sclerosis (Jiménez-García et al., 2024), Alzheimer’s (Maruthiyodan et al., 2024), coronary artery disease (Jia et al., 2019), and Parkinson’s (Sena-dos-Santos et al., 2024) have already been associated with changes in mitochondrial function.

In recent years, the Coronavirus Disease 2019 (COVID-19) has also been closely related to mitochondrial dysfunction due to the infection process by the Severe Acute Respiratory Syndrome-Coronavirus 2 (*SARS-CoV-2*) associated with mitochondrial membrane depolarization, opening of the mitochondrial permeability transition pore, increased release of reactive oxygen species (ROS), and mitophagy impairment (Shang et al., 2022). These processes can lead to stimulating disruption of immune function and unbalancing immune and pro-inflammatory responses (Moreno Fernández-Ayala et al., 2020; Tereshin et al., 2022; Shoraka et al., 2023a), since mitochondrial morphological and functional changes occurs during *SARS-CoV-2* infection. 

The pandemic caused by the coronavirus known as *SARS-CoV-2* had a significantly profound impact on economic, social and medical systems, resulting in a global health emergency, the consequences of which still affect certain countries (Moreno Fernández-Ayala et al., 2020; Scozzi et al., 2021).

COVID-19 presents a wide clinical spectrum, ranging from asymptomatic or mild cases (flu-like symptoms) to severe cases of respiratory failure and death. While advanced age, male sex, and some comorbidities are known risk factors, they do not fully account for this spectrum, since young individuals without preexisting conditions were observed experiencing the severe form of the disease, often characterized by low oxygen saturation and inflammatory lung responses (Andolfo et al., 2021; Brodin, 2021; O’Driscoll et al., 2021; Ochani et al., 2021; Angulo-Aguado et al., 2022). These features suggest that severe COVID-19 appears to be associated with a dysregulated immune and pro-inflammatory response, in which mitochondria play an important role (Shoraka et al., 2023a). 

When this state of inflammation, endothelial damage, and mitochondrial dysfunction persists, the risk of developing complications associated with the Post COVID-19 condition (PCC) increases. Mitochondrial dysfunction related to Post COVID-19 symptoms contribute to persistent fatigue, muscle weakness, exercise intolerance, post-exertional malaise, cognitive impairments, cardiovascular abnormalities, and shortness of breath. Consequently, these issues are linked to deficits in energy production, impaired immune response, metabolic disturbances, and endothelial and vascular dysfunction (Georgieva et al., 2023; Molnar et al., 2024).

In this review, we describe the role of mitochondria in modulating the immune response against *SARS-CoV-2*, the mechanisms of subversion of these organelles by the virus, the influences of the mitochondrial genome on susceptibility and the maintenance of its dysfunction associated with the development of Post COVID-19 Condition.

## Methodology

To address the objectives of this study, a systematic literature search was conducted across the PubMed, Web of Science, and Google Scholar databases. The search strategy was designed to capture studies investigating the relationship between mitochondria and *SARS-CoV-2* infection, as well as its potential impact in the Post COVID-19 condition. Eligibility criteria were defined prior to the selection process. Studies were included if they were published in English, available in full-text format, and classified as either original research articles or review papers. Exclusion criteria comprised studies without full-text availability, conference abstracts, editorials, letters to the editor, and publications outside the established time frame.

The search was performed using a combination of relevant keywords, including: mitochondria, mitochondrial genome, mtDNA variants, mitochondrial mechanisms, *SARS-CoV-2*, COVID-19, Post COVID-19, innate immune response, neurological sequelae, cardiac sequelae, muscular sequelae, mitochondrial dysfunction and therapeutic approaches of Post COVID-19 condition. These terms were applied individually and in combination to maximize the retrieval of relevant studies. The selection process was conducted in two stages. First, articles were screened based on titles and abstracts to identify potentially relevant studies. Subsequently, full-text assessments were performed to confirm eligibility according to the predefined inclusion and exclusion criteria. This approach ensured a rigorous and comprehensive evaluation of the available literature.

## General characteristics of human mitochondria

Mitochondria are essential cytoplasmic organelles that participate in important processes in cellular function and operation, including cell death, control of calcium levels, lipid homeostasis, metabolic cell signaling and generation of about 90% of cellular energy in the form of Adenosine Triphosphate (ATP), mainly by OXPHOS, but also by the tricarboxylic acid cycle (TCA) (Yan et al., 2019; Cavalcante et al., 2019).

Structurally, a mitochondrion is divided into an outer membrane and an inner membrane, and the spaces generated by these membranes define two distinct compartments: the intermembrane space (IMS) and the mitochondrial matrix, which is where TCA occurs and the mitochondrial genome (mitogenome) is located (Mitchell et al., 2014; Nguyen et al., 2020; Oliveira et al., 2021). The outer membrane is smooth, highly permeable, and only slightly selective for cytosolic solutes (Nguyen *et al.,* 2020; Oliveira *et al.,* 2021). The inner membrane, on the other hand, is folded into structures called cristae, and is less permeable and highly selective for solutes, also carrying the system responsible for OXPHOS, known as the electron transport chain (ETC) (Cavalcante et al., 2020; Nguyen *et al.,* 2020; Oliveira *et al.,* 2021).

### Mitochondrial genome

**Figure 1 -:**
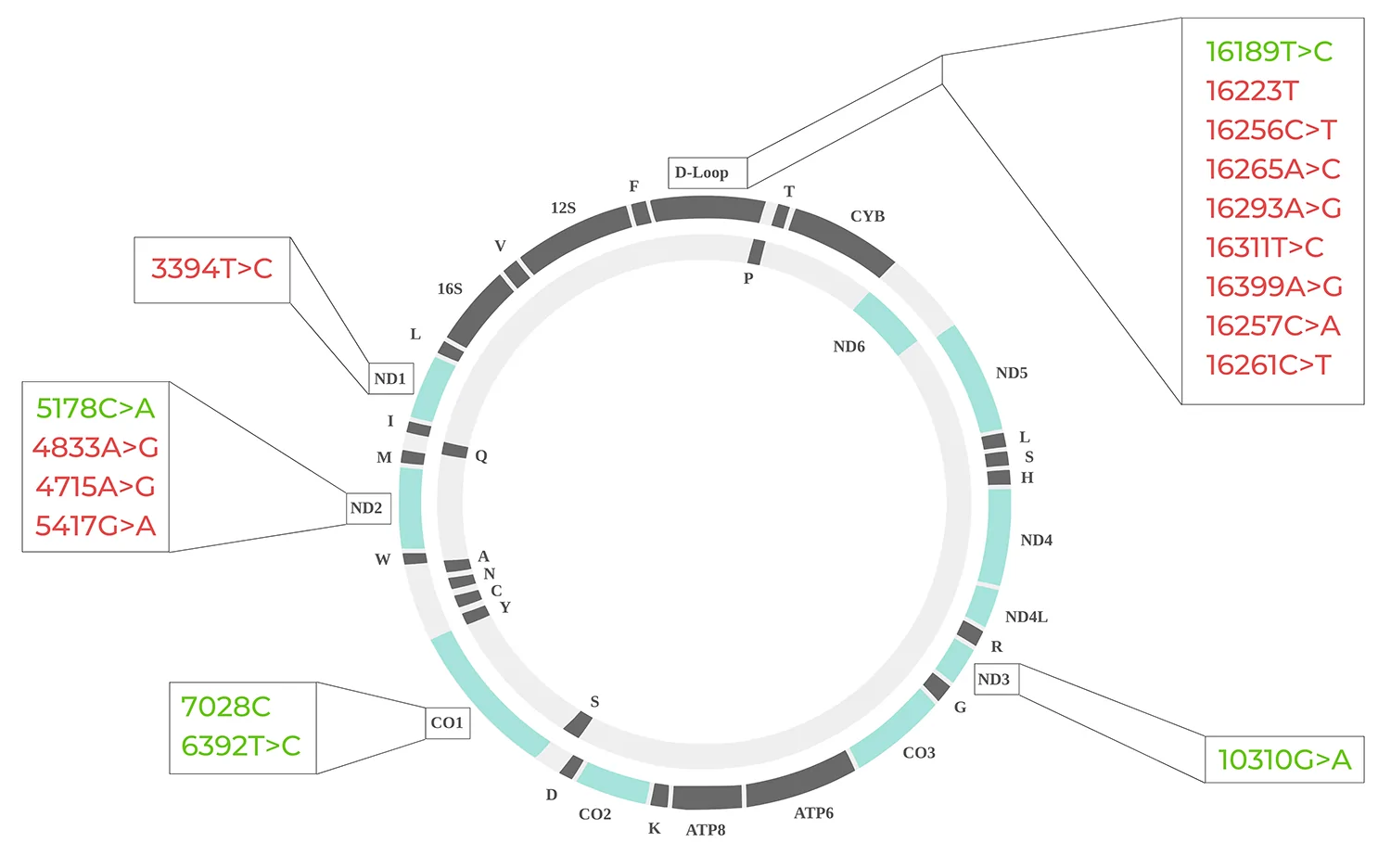
Mitochondrial genome and the variants already associated with severe COVID**.** Protein-coding genes are represented in light blue, while other regions are portrayed in gray. In red, there are the mitochondrial variants associated with an increased risk of developing the severe form of covid and, in green, those associated with a protective effect. Source: Authors (created with Inkscape v.1.4.3).

The human mitogenome is organized into a circular double-stranded DNA (mtDNA) and is composed of 16,569 bp (Roger et al., 2017; Nissanka and Moraes 2020; Chapman et al., 2020; Pérez-Amado et al., 2021). Such genome is structured into a heavy strand (H-strand) and a light strand (L-strand), in which the H-strand is rich in guanine and encodes 28 genes and the L-strand is rich in cytosine and encodes nine genes (Roger *et al.,* 2017; Nissanka and Moraes 2020; Chapman *et al.,* 2020; Pérez-Amado *et al.,* 2021). The coding region of mtDNA contains 37 genes, 13 of which are genes encoding polypeptides associated with OXPHOS, 22 transfer RNA (tRNA) genes and two ribosomal RNA (rRNA) genes, seen in Figure 1 (Badano et al., 2018; Nissanka and Moraes 2020; Pérez-Amado *et al.,* 2021).

Previous studies indicate that mutations in mitochondrial genes can disrupt key cellular functions, including synaptic integrity, axonal maintenance, ROS regulation, and mitophagy (Bergman and Ben-Shachar 2016; Andrieux et al., 2021). These disruptions contribute to the development of various complex diseases, such as cancer, neurodegenerative disorders, and infectious diseases (Prates Mori and de Souza-Pinto 2018; Roca-Bayerri et al., 2021; Stanke et al., 2021), such as COVID-19. Figure 1 also shows variants that have already been associated with the risk and protection of developing severe COVID-19.

After analyzing mtDNA from 316 patients who required admission to the intensive care unit due to COVID-19, Vázquez-Coto et al. (2022) identified the 7028C with a protective effect and the 16223T variant as a risk factor for the development of the critical course of this disease (Vázquez-Coto *et al.,* 2022). A study in a Chinese population identified mtDNA variants in the D-loop region associated with an increased risk (4833A>G, 4715A>G, 3394T>C and 5417G>A, 16257C>A, 16261C>T) and with an individual resistance (249delA, 6392T>C, 10310G>A) (Wu et al., 2021). In addition, Bľandová et al (2024) observed 16256C>T, 16265A>C, 16293A>G, 16311T>C and 16399A>G variants with a higher risk for a severe course of COVID-19 and 16189T>C with a protective effect towards it (Bľandová *et al.,* 2024).

### Mitochondrial recognition of *SARS-CoV-2*

Mitochondria can be extremely vulnerable to physiological and pathological stimuli, such as viral infections. Depending on the pathogen, different stimuli are applied to mitochondria, which may cease performing their functions normally. Regarding viral pathogens, mitochondria play an essential role in host antiviral signaling through the mitochondrial antiviral signaling protein (MAVS), located on their outer membrane (Shoraka et al., 2023a). Following the virus entry into the host cell, viral RNA is first recognized by the host’s pattern recognition receptors (PRRs) (such as RIG-I and MDA5 receptors) and initiates the receptor signaling pathway and activates immune responses dependent on MAVS (Su et al., 2022; Shoraka *et al.,* 2023a).

MAVS acts as a platform for signaling downstream of PRRs and can mediate the activation of NF-κB and interferon regulatory factor 3 and 7 (IRF3/7). This activation subsequently promotes the expression of several pro-inflammatory cytokines and the induction of antiviral genes, such as type I interferon (IFN-I) and IFN-stimulated genes (ISGs), which prevent viral replication and transmission (Su et al., 2022; Chang et al., 2022; Shoraka et al., 2023a).

Among the evasion pathways from MAVS by *SARS-CoV-2*, we highlight the formation of double membrane vesicles (DMV) through the host’s mitochondria and endoplasmic reticulum around its double-stranded RNA intermediate, thus protecting it from detection (Shenoy, 2020; Valdés-Aguayo et al., 2021). These vesicles, in addition to being appropriate sites for replication, prevent the host from digesting the virus (Valdés-Aguayo *et al.,* 2021).

The *SARS-CoV-2* genome is characterized by accessory proteins called open reading frames (ORF), which interact with receptors on the mitochondrial outer membrane, limiting the host cell’s initial response (Shenoy 2020). Studies demonstrate that, in COVID-19, there is a decrease in the production of type I interferons IFN-I due to mitochondrial exposure to *SARS-CoV-2* proteins, probably through the interaction of ORF9b with poly (C) binding protein 2 (PCBP2) and AIP4 (an E3 ubiquitin protein ligase) (Denaro et al., 2022; Shoraka et al., 2023b). 

Furthermore, some studies have found that ORF10 can induce the degradation of mitochondria through mitophagy by targeting MAVS and the INF-I signaling pathway (Denaro et al., 2022) and ORF9c of *SARS-CoV-2* can also interact with the production of negative regulators of MAVS signaling (NLRX1, NDFIP2), thus affecting mitochondrial function (Chang et al., 2022).

For example, ORF3a, in addition to mediating mitochondrial apoptosis, also targets the mitochondrial deubiquitinase Ubiquitin-Specific Protease 30 (USP30), thus altering mitochondrial homeostasis and quality control. Furthermore, it likely plays a role in the activation of hypoxia-inducible factor-1α (HIF-1α) that enhances viral infectivity (Nunn et al., 2022; Chang et al., 2022). It has been suggested that one of the strategies used by viruses, including *SARS-CoV-2*, to evade the host’s immune system involves modifying the dynamics of mitochondrial genes (Burtscher et al., 2020). 

**Figure 2 -:**
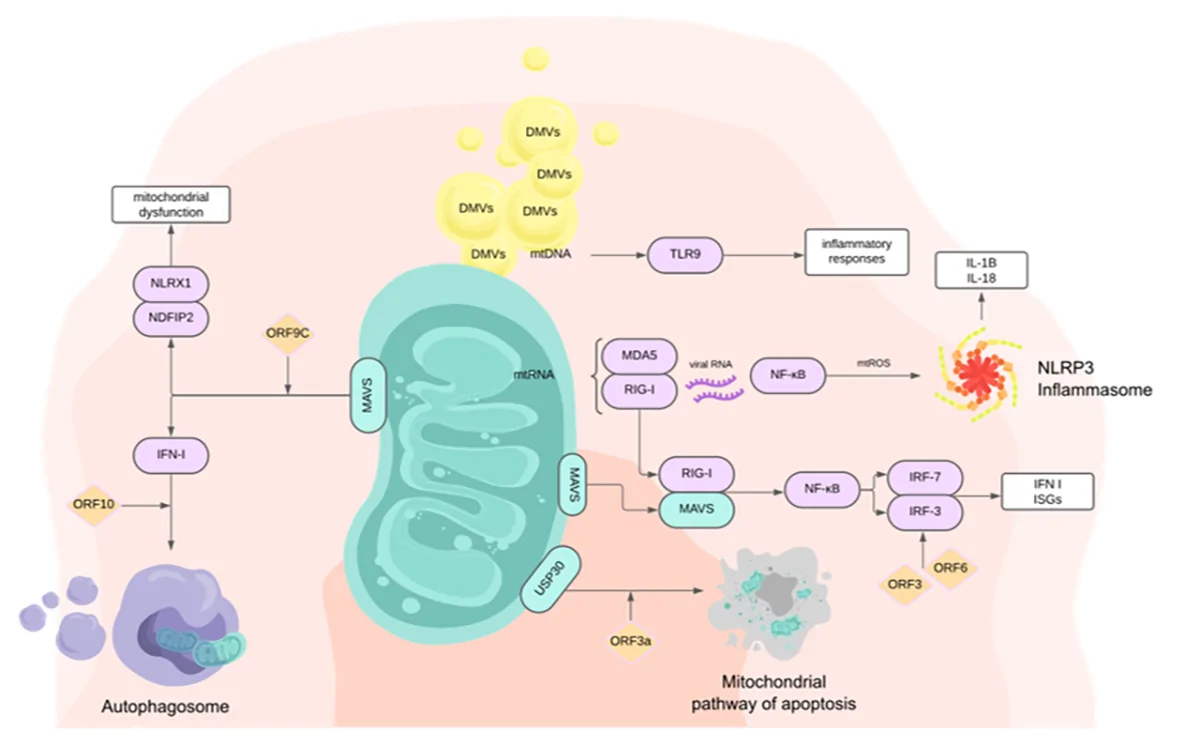
Mechanism of mitochondrial damage and innate immune dysregulation caused by the *SARS-CoV-2* infection. In normally functioning cells, various mechanisms exist to warn of mitochondrial damage and viral infection, most notably via retinoic acid inducible gene-1 and mitochondrial antiviral signalling protein (RIG-1-MAVS), Toll-like receptor-9 (TLR9), Ubiquitin Specific Peptidase 30 (USP30) and activation of the NOD-, LRR -and pyrin domain containing protein 3 (NLRP3) inflammasome. Abbreviations: DMVs-double membrane vesicles; IL-1β-interleukin 1 beta; IL18-interleukin 18; IFN I-type I interferon; IRF3- interferon regulatory factor 3; IRF7- interferon regulatory factor 7; ISGs-IFN-stimulated genes; MAVS-mitochondrial antiviral-signalling protein; MDA5- melanoma differentiation-associated protein 5; NFκB-nuclear factor kappa-light-chain-enhancer of activated B cells; NSP-non-structural protein, ORF-open reading frame; mtDNA-mitochondrial DNA; mtROS-reactive oxygen mitochondrial species; NLRX1- NLR Family Member X1; NDFIP2- Nedd4 Family Interacting Protein 2; TLR9-Toll-like receptor 9; USP30- Ubiquitin Specific Peptidase 30. Orange objects indicate material of viral origin, purple and green objects indicate self-material, being nuclear and mitochondrial, respectively. Source: Authors (created with Inkscape v.1.4.3).

Specific toll-like receptors (TLRs) expressed in endolysosomal compartments are also PRRs and participate in viral identification. The coronavirus spike protein, which is responsible for preventing apoptosis during viral replication within the cell, is recognized by TLR2/4, and the single-stranded RNA (ssRNA) is detected by TLR7/8 (Kalashnyk et al., 2021; Valdés-Aguayo et al., 2021; Su et al., 2022). TLRS sensing leads to the recruitment of adapter proteins, which activate NF-κB signaling and promote the expression of interleukin (IL) precursors and an increase in the expression of NADPH oxidase (NOX), followed by ROS production (Su *et al.,* 2022). Figure 2 illustrates the key mechanisms previously mentioned of mitochondrial damage and innate immune dysregulation induced by *SARS-CoV-2* infection.

### Mitochondrial genetic dysfunction due to *SARS-CoV-2*

The recognition of *SARS-CoV-2* by mitochondria causes several dysregulations, such as excessive production of ROS and morphological damage, such as swelling and changes in the size and number of these organelles, which can prevent mitochondrial dynamics and biogenesis (Chang et al., 2022; Molnar et al., 2024).

### Damage-associated molecular patterns (DAMPs) and mtDNA

Apart from general cellular dysfunctions, exposure to *SARS-CoV-2* can even induce variations in the number of copies of mitochondrial genes. This is due to mitochondria having several potent immunostimulatory damage-associated molecular patterns (DAMPs) which, like pathogen-associated molecular patterns (PAMPs), are recognized by PRRs and activate the immune system upon exposure to the cytosol or release into the extracellular environment in response to stress and loss of homeostasis (Scozzi et al., 2021; Souza and Cavalcante 2022; Shoraka et al., 2023b).

Although mitochondrial DAMPs are important elements in the immune response, their over-detection can trigger an undesirable pro-inflammatory response. In conditions of massive cellular damage, such as COVID-19, mitochondrial protective mechanisms, as well as the mitophagy processes in which damaged mitochondria are phagocytosed, can become overwhelmed or dysfunctional, inducing the release of mitochondrial DAMPs into the cytosol and circulation in overwhelming quantities (Valdés-Aguayo et al., 2021). 

When *SARS-CoV-2* is in contact with the mitochondria, it forms double-membrane vesicles and releases mtDNA, a DAMP itself, into circulation, which, in turn, leads to the initiation of inflammatory responses by being recognized by the *TLR9* gene because of methylated CpG motifs (Scozzi et al., 2021; Valdés-Aguayo et al., 2021). 

Based on this, it is possible to hypothesize that mtDNA can be used as an indicator of the acute severity of COVID-19, as positive correlations have been observed between its levels and pro-inflammatory markers, such as lactic acid dehydrogenase (LDH) and D-dimer levels, in the plasma of COVID-19 patients (Scozzi et al., 2021; Georgieva et al., 2023).

Importantly, release of mtDNA is often accompanied by the release of other MT-DAMPs, such as N-formylated peptides, cytochrome c, and cardiolipin, which collectively can induce the expression of inflammatory cytokines and the generation of ROS, which may directly contribute to acute lung injury and systemic inflammation (Scozzi et al., 2021).

*Activation of the NLRP3 Inflammasome Pathway*

The *NLRP3* inflammasome pathway plays a key role in the innate immune response against pathogens, including transcriptional activation of *NLRP3* and pro-inflammatory cytokines, such as pro-IL-1β and pro-IL-18, in response to inflammatory molecular patterns (PAMPs) and inflammatory markers (DAMPs) (Blevins et al., 2022). Initiation signals can be mediated by several receptors, including Toll-like receptors (TLRs), which activate nuclear factor B (NF-κB) to stimulate gene expression (Li et al., 2024). 

The second signal is triggered by several PAMPs and DAMPs, leading to *NLRP3* oligomerization and inflammasome complex assembly. The activation of the inflammasome can be influenced by multiple factors, including potassium efflux, calcium flux, mitochondrial reactive oxygen species (mtROS) production, lysosomal damage, and release of oxidized mitochondrial DNA (Blevins et al., 2022; Zhan et al., 2023).

In the *SARS-CoV-2* infection, the increased activation of the *NLRP3* inflammasome is associated with acute lung inflammation and acute respiratory distress syndrome (ARDS), frequently observed in severe cases of the disease and responsible for most deaths (Freeman and Swartz 2020; Pan et al., 2021).

*Cytokine storm and activation of the cGAS-STING pathway*

The mitochondrial genome also has important functions as a mediator of the antiviral immune response, such as promoting inflammation through immune pathways such as cyclic GMP-AMP synthase stimulator of interferon genes (cGAS-STING) signaling, activating inflammasomes, and releasing cytokine storms (Shoraka et al., 2023b). 

**Figure 3 -:**
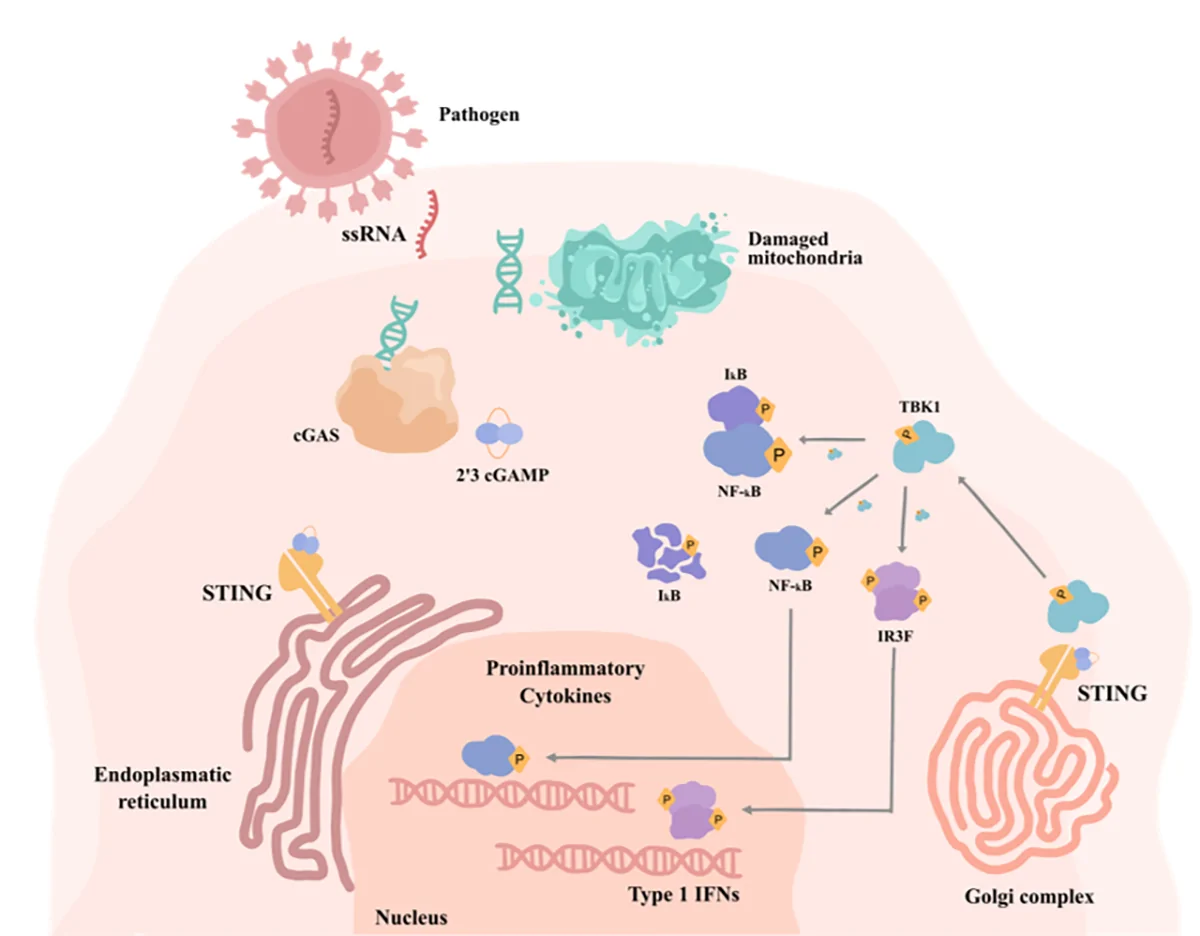
Activation of the cGAS-STING pathway and its pro-inflammatory immune response. The cGAS binds to double-stranded DNA (dsDNA), producing 2’3’-cGAMP, which activates STING. This induces a conformational change, forming STING tetramers and triggering its translocation from the ER to the Golgi. Modified STING recruits TBK1 and IRF3 and stimulates IkB, activating NF-kB transcription. This regulates the expression and secretion of pro-inflammatory cytokines. Source: Authors (created with Inkscape v.1.4.3).

The cGAS-STING signaling pathway regulates IFN-I induction on exogenous and endogenous DNA and is activated by host cell stress induced by viral infection and dsDNA (Kwon et al., 2017; Su et al., 2022). cGAS dimers are assembled upon binding to dsDNA, leading to activation of the cGAS enzyme and synthesis of 2’,3’ cyclic GMP-AMP (cGAMP) (Su *et al.,* 2022). The binding of cGAMP to the STING dimer induces a significant conformational change at its C-terminus, significantly alters its conformation, and triggers STING oligomerization (Kwon *et al.,* 2017; Su *et al.,* 2022). Figure 3 illustrates the activation and regulation processes of the cGAS-STING pathway.

Studies have reported that severe COVID-19 appears to be associated with a dysregulated immune response and the release and amplification of a cytokine storm, which leads to a hyperinflammatory state (Valdés-Aguayo et al., 2021; Shoraka et al., 2023a). Cytokine storm is triggered by a significant ROS increase, resulting in the release of TNF-a, IL-1β, IL-6 and IL-18, which act as mediators and amplifiers of inflammation, promoting the activation of the inflammasome. As a consequence of this activation, cells reprogram their metabolism, intensifying glycolysis and reducing ATP production via TCA, which leads to mitochondrial atrophy (Valdés-Aguayo *et al.,* 2021). 

*Synergy between the cGAS-STING pathway and inflammasomes*

The cGAS-STING pathway interacts synergistically with inflammasomes, such as *NLRP3*, to amplify immune responses. Cytosolic mtDNA activates cGAS, leading to the production of cGAMP and subsequent activation of *STING*. This activation triggers the phosphorylation of transcription factors such as IRF3 and NF-κB, promoting the production of type I interferons (IFN-α and IFN-β) and inflammatory cytokines (Kim et al., 2023; Zhou et al., 2023).

The genes responsible for the cGAS-STING pathway include *CGAS* or *MB21D1*, located in chromosome 6, and *STING1* or *TMEM173*, located in chromosome 5. As expected, these genes may influence immune responses to infections. For example, specific single-nucleotide polymorphisms (SNPs) in *TMEM17*3 have been associated with altered IFN-α production in response to viral stimuli, potentially affecting susceptibility to infections (Patel and Jin 2019; Kennedy et al., 2020).

*Mitochondrial regulation of mitophagy and apoptosis pathways*

The *PINK1* and *PRKN* genes play crucial roles in mitochondrial regulation through the process of PINK1/Parkin mitophagy, which is the selective degradation of damaged mitochondria. This occurs with the recruitment of the *PINK1* and Parkin proteins, which signal these mitochondria for degradation after the formation of the mitophagosome (compartment that retains mitochondria for degradation). In this way, lysosomal enzymes degrade the inner membrane of the mitophagosome and its contents (Ma et al., 2024).

The mitophagy process eliminates damaged mitochondria, preventing them from releasing inflammatory signals, and several microorganisms, such as *SARS-CoV-2,* interfere with it, resulting in damaged mitochondria and accumulation of autophagosomes. This process eliminates damaged mitochondria, preventing them from releasing inflammatory signals (Wang et al., 2023). The genes *ATG5* and *BECN1* are also crucial for mitophagy and autophagy in general since they facilitate the removal of damaged cellular components, including compromised mitochondria, limiting the release of ROS and mtDNA that could activate exaggerated inflammation. Autophagy mediated by these genes helps control the inflammatory response, preventing tissue injury and promoting homeostasis during infections (Menon and Dhamija, 2018).

The *BCL2* and *BAX* genes regulate mitochondrial apoptosis. *BCL2* inhibits apoptosis and protects mitochondria under normal conditions, while *BAX* promotes apoptosis when activated by cellular stress or infection. The balance between *BCL2* and *BAX* is essential to eliminate infected or damaged cells without triggering excessive inflammation. Therefore, controlled apoptosis is an effective way to contain the spread of the pathogen in response to viral infections (Warren et al., 2019).

During *SARS-CoV-2* infection, the spike protein was found to regulate the expression of both *BCL2* and *BAX*. This regulation leads to changes in mitochondrial outer membrane permeability (MOMP), resulting in the release of cytochrome c from the mitochondria into the cytoplasm. This release is a key step in the activation of caspases, leading to apoptosis (Yuan et al., 2023). 

## Post COVID-19 Condition (PCC)

If the mitochondrial dysfunctions described above persist without adequate recovery, individuals may experience chronic cellular injury and develop various clinical sequelae. Post COVID-19 condition, also known as post-acute sequelae of COVID-19, can be defined as a diversity of continuous, recurrent or new symptoms lasting from two to six weeks, depending on the clinical degree of *SARS-CoV-2* infection, that are not explained by other causes, and affects approximately 50-80% of previously symptomatic patients with COVID-19 (Raveendran 2021; Nunn et al., 2022; Cao et al., 2023; Noonong et al., 2023; Mantle et al., 2024). Although most individuals recover from a COVID-19 infection within a few weeks, some individuals continue to experience prolonged symptoms that can significantly affect their daily functioning and quality of life (Nunn *et al.,* 2022; Mantle *et al.,* 2024). 

The PCC has long-term heterogeneity and complex symptoms, but the most common symptomatology of this new disease includes fatigue, followed by depression, anxiety, cognitive dysfunction, sleep disorders, shortness of breath, loss of smell/taste, cough, joint pain, chest pain, muscle pain and headaches (Nunn et al., 2022; Noonong et al., 2023).

Among the risk factors for post COVID-19 condition, there are advanced age, female sex, comorbidities such as obesity, diabetes, pre-existing respiratory disorders and the clinical severity of the *SARS-CoV-2* infection (Visco et al., 2022; Mantle et al., 2024). However, it remains unclear why some individuals develop PCC while others fully recover after an acute COVID-19 (Mantle *et al.,* 2024), being speculated that dysregulated immune responses, *SARS-CoV-2*-specific pathophysiology and its permanence in certain tissues and inflammatory damage in response to acute infection are involved in this process, especially mitochondrial dysfunction (De Melo et al., 2021; Nalbandian et al., 2021; Chen et al., 2023a).

## Post COVID-19 condition and mitochondrial dysfunctions

As previously mentioned, mitochondrial dysfunctions related to COVID-19 include increased oxidative stress, impaired cellular energy production and the exacerbation of inflammatory responses (Molnar et al., 2024). These consequences can be observed in PCC symptoms, such as cognitive difficulties, fatigue and muscle weakness, shortness of breath, and heart issues that can possibly occur from mitochondria-related mechanisms as energy production deficits, immune system imbalances, metabolic disruptions and vascular and endothelial dysfunction (Madsen et al., 2024; Molnar *et al.,* 2024). 

For instance, when analyzed the O_2_ consumption rates of the peripheral blood mononuclear cells (PBMCs) in patients with lower physical activity level after COVID-19 infection, Silva et al. (2023) observed that the mitochondrial production of ATP by PBMC decreases, suggesting that these cells are relying on glycolytic metabolism or using alternative substrates for mitochondrial respiration, such as fatty acids (Silva *et al.,* 2023). 

Metabolomic and transcriptomic studies have already established that SARS-CoV-2 reprograms the host’s metabolism to favor fatty acid oxidation (FAO) over glucose metabolism in order to increase viral replication (Bruzzone et al., 2020; Nie et al., 2021). This process increases plasma free fatty acid levels, especially poly- and highly unsaturated fatty acids, and causes a reduction in beta-oxidation and an increase in ROS production, which are signs of mitochondrial dysfunction (Molnar et al., 2024). This can result in lactate accumulation, generating intolerance to more intense physical exercise, as previously reported (Guntur et al., 2022).

Moreover, mitochondrial damage can prevent the breakdown of lipids, leading to their storage in tissues such as the liver and adipose tissue. In turn, this can exacerbate inflammation and insulin resistance (Madsen et al., 2024). Furthermore, just as mitochondrial dysfunction alters lipid metabolism, lipid dysregulation can also disrupt mitochondrial homeostasis. It has been observed that lipid mediators such as arachidonic acid are elevated in post-Covid condition patients, activating NLRP3 inflammasomes and perpetuating mitochondrial ROS production (Lucena Lage et al., 2025). In addition, Bizjak et al. (2024) observed that physiological differences such as lower complex I activity and a higher intermyofibrillar cristae integrity of the mitochondria in post COVID-19 condition patient’s muscle biopsy when compared to age-matched healthy controls (Bizjak *et al.,* 2024). 

Such physiological and cellular mitochondrial damage observed may occur due to the condition that *SARS-CoV-2* can remain in infected tissues, such as lung and nasopharyngeal cells, even after the viral load is eliminated, as observed by Guarnieri et al. (2023) in autopsied tissues. Besides this, the research group also observed that OXPHOS genes, both nDNA- and mtDNA-coded, are transcriptionally inhibited during acute COVID-19, resulting in increased mitochondrial reactive oxygen species (mROS) production. This increase in mROS, in parallel with ensuring viral biogenesis, induces the release of mtDNA that acts as mtDAMPs activating the NLRP3-inflammasome and other immune system genes such as *TLR9* and *IFN-1* (Zhong et al., 2018; Guarnieri *et al.,* 2024).

When investigating physiological patterns after one year from initial *SARS-CoV-2* infection, Peppercorn et al. (2023) observed 21 differentially regulated mitochondrial proteins involved in metabolism, translation, dynamics, and OXPHOS (Peppercorn *et al.,* 2023).

These mitochondrial changes may permanently alter the epigenome of individuals by suppressing OXPHOS gene expression even after viral genomes have been eliminated, as already observed in heart autopsies of COVID-19 patients, where nDNA OXPHOS genes remained silenced, and in hamster olfactory epithelial cells, where olfactory receptor gene expression persisted inhibited (Zazhytska et al., 2022; Guarnieri et al., 2023). Then, it is possible to hypothesize that the inhibition of OXPHOS gene expression may be associated with the persistence of the damage caused by the *SARS-CoV-2* infection that evolves into the symptoms observed in the post-COVID syndrome.

In this context, increased levels of Cytochrome c Oxidase subunit 7A1 (COX7A1), a protein part of the mitochondrial respiration chain responsible for oxidative phosphorylation, were found in patients with neurological sequelae when compared to controls and COVID-19 convalescents. When analyzing specific neurologic symptoms, COX7A1 expression was significantly higher in patients with numbness, pain and fatigue (Hanson et al., 2023).

As mentioned earlier, fatigue is one of the most common symptoms post COVID-19 condition and can be caused by dysfunctions in muscle oxidative capacity, which in turn can be due to alterations in mitochondrial homeostasis. When analyzing *vastus lateralis* muscle samples, Colosio et al. (2023) observed lower levels of the pro-fusion protein OPA1 and PGC1α, a key regulator of mitochondrial biogenesis, and higher levels of pro-fission activated by cellular stress proteins DRP1 and FIS1 (Colosio *et al.,* 2023). Moreover, elevated levels of DRP1, along with mitochondrial outer membrane fusion protein MFN2, were also observed by Szögi et al. (2024) in PCC patients (Szögi *et al.,* 2024). 

This imbalance in proteins that regulate mitochondrial fusion and fission could indicate the cell’s attempt to maintain its homeostasis. However, these pro-fission shifts can, apart from not returning mitochondria to their normal state, increase the chances of a permanent imbalance occurring in the metabolism of cellular energy, leading to the development of symptoms such as chronic fatigue and muscular weakness (Colosio et al., 2023; Szögi et al., 2024).

Is the reason for the alteration in the levels of these proteins only due to occasional/environmental factors or are intrinsic and genetic host factors behind this? Is it possible that these individuals already had mutations in genes that are essential for mitochondrial functioning and would develop symptoms, such as those mentioned above, throughout their lives and the *SARS-CoV-2* infection only accelerated this progress? Or did the sequelae only develop because of contact with the virus? Unfortunately, it is still not possible to answer these questions due to the scarcity of studies on mitochondrial genetic variants and their direct correlation with the development of post COVID-19 sequelae.

Meanwhile, in parallel with proteins, some biochemical markers associated with oxidative stress, such as the antioxidants coenzyme Q10 and Serum peroxiredoxin-3, have already been observed as contributing factors to mitochondrial dysfunction in PCC (Karpenko et al., 2021; Sumbalova et al., 2021). 

## Mitochondria in cardiac and muscular sequelae

One of the hallmark symptoms of PCC is chronic fatigue, which can be directly linked to defects in mitochondrial ATP production. When mitochondrial function is compromised, ATP production is reduced, leading to energy deficits that manifest as profound and persistent fatigue (Komaroff and Lipkin, 2023; Molnar et al., 2024). This energy shortfall impacts muscle function. Fatigue in PCC bears resemblance to the fatigue experienced by heart failure patients, a condition where mitochondrial dysfunction has also been implicated (Kedor et al., 2022; Gil et al., 2023; Appelman et al., 2024). In heart failure, compromised mitochondrial efficiency significantly impairs cardiac function, directly contributing to symptoms of fatigue and limited exercise capacity (Zhou and Tian, 2018). Given the heart’s reliance on OXPHOS for its substantial energy needs, any reduction in this process can result in marked energy deficits. This mitochondrial inefficiency in heart failure parallels the potential role of mitochondrial dysfunction in exacerbating the fatigue observed in PCC patients (Rosca and Hoppel, 2013; Li *et al.,* 2023; Gallo et al., 2024).

Mitochondrial dysfunctions may also predispose individuals to myopathies and cardiomyopathies, which constitute progressive muscular conditions and are primarily caused by failures in OXPHOS mechanisms (Bottoni et al., 2022; Chang et al., 2022; Mantle et al., 2024). In PCC patients, cardiovascular complications include myocarditis, myocardial injury, microvascular injury, pericarditis, acute coronary syndrome, and arrhythmias. These complications are closely linked to mitochondrial dysfunction induced by *SARS-CoV-2*, as mitochondria serve as the energy center for cardiomyocytes. When dysfunctional, they release their contents into the cytoplasm, triggering the production of inflammatory cytokines and the activation of apoptotic pathways. This process leads to senescence and cell death of cardiomyocytes, exacerbating cardiovascular conditions in patients with post COVID-19 condition (Chang *et al.,* 2022).

In addition, it is known that some mtDNA variants, such as 3260A>G, 3303C>T, 4320C>T and 4300A>G may directly affect mitochondrial function and facilitate the onset of post-COVID sequelae in cardiac and other muscle tissues, influencing conditions like the mitochondrial myopathy and cardiomyopathy (Lott et al., 2013; Nunn et al., 2022; Yang et al., 2022; Madsen et al., 2024).

The impact of mtDNA variants on pathological conditions adds a layer of complexity to the understanding of mitochondrial dysfunctions. The mitochondrial variant 3260A>G has been associated with various clinical manifestations, including mitochondrial myopathy, MELAS (mitochondrial encephalomyopathy, lactic acidosis, and stroke-like episodes), maternal myopathy, and cardiomyopathy, as well as exercise-induced rhabdomyolysis (Connolly et al., 2010).

Similarly, the 3303C>T variant may be associated with mitochondrial myopathy and cardiomyopathy, as well as deficiencies in respiratory chain enzymes. Heteroplasmic mutations in the tRNA^Leu(UUR) gene impair mitochondrial protein synthesis, which is essential for the proper functioning of muscular and cardiac tissues (Iwanaga et al., 2001). The 4320C>T variant, in turn, is linked to several pathologies, particularly cardiomyopathies (Levinger, 2003). Notably, this variant was found in genomic patterns of *SARS-CoV-2* samples collected in Macaé, Brazil, suggesting potential interactions between mitochondrial mutations and viral infections (da-Costa-Rodrigues et al., 2022). Finally, the 4300A>G variant also plays an important role in mitochondrial cardiomyopathy, being associated with hereditary conditions such as maternal hypertrophic cardiomyopathy, as well as dysfunctions in the mitochondrial respiratory chain in cardiac tissues (Chen et al., 2023b).

## Mitochondria in neurological sequelae

In addition to muscle tissues, the brain presents a significant concentration of mitochondria. Due to the high expression of the angiotensin-converting enzyme 2 (ACE2) receptor in various regions of the brain, *SARS-CoV-2* is also capable of causing mitochondrial dysfunction in nerve cells, causing acute brain damage and also underlining the development of subsequent, and potentially long-term, neurodegenerative changes (Lukiw et al., 2022).

Neural mitochondrial dysfunctions triggered by *SARS-CoV-2* further exacerbate neurological symptoms, as neurons have high ATP demands (Esch et al., 2002). In many cases, the neural sequelae of COVID-19 may share mitochondrial dysfunction markers, such as neuroinflammation, increased apoptosis, and elevated ROS generation (Denaro et al., 2022).

Large evidence supports that *SARS-CoV-2* induces neurological alterations, primarily due to its ability to cross the blood-brain barrier and invade neural cells. These sequelae include migraines, frequent memory lapses, and inflammatory processes in neural tissues, potentially leading to additional neurological disorders (Pezzini and Padovani, 2020). For instance, the 1606G>A variant has been linked to a spectrum of neurological impairments, including progressive ataxia, seizures, cognitive decline, mild myopathy, and hearing loss (Ding et al., 2023). 

Beyond that, the 3256C>T variant has been associated with the formation of atherosclerotic plaques, which can contribute to vascular complications such as stroke (Sobenin et al., 2012). These variants play a critical role in disease pathogenesis, particularly in conditions involving mitochondrial dysfunction, underscoring the impact of mtDNA alterations on cellular energy metabolism and disease progression (Lott et al., 2013; Liu et al., 2021; Ng and McFarland, 2023).

Several other mtDNA variants have been previously linked to various health conditions. For instance, the 7443A>G, 1494C>T, 7510T>C and 7444G>A substitutions have been associated with deafness and sensorineural hearing loss, a condition for which the underlying etiology remains largely unknown (Postal et al., 2009; Tripathi and Deshmukh, 2022). These variants may play a role in mitochondrial dysfunction, potentially affecting auditory cells and contributing to hearing impairment.

The 3196G>A also exhibits a particularly intriguing correlation with pediatric brain tumors. However, beyond its association with oncological conditions, this variant is found within haplogroups linked to neurodegenerative diseases, such as Alzheimer’s and Parkinson’s (Luna et al., 2015). This dual association suggests a broader impact of mitochondrial variants on neurological health, warranting further investigation into their potential mechanisms and implications.

Furthermore, it is important to recognize that individuals from different geographic regions carry distinct genetic backgrounds, which may influence the prevalence and severity of mitochondrial dysfunctions and related conditions. Understanding these regional genetic variations is essential for advancing personalized medicine, as it can guide researchers and clinicians in tailoring more effective prevention strategies and interventions to mitigate the risk of severe health outcomes within specific populations (Bergström et al., 2020; Hünemeier, 2024).

## Therapeutic approaches and future perspectives

Regarding post COVID-19 condition, the multifaceted etiopathogenesis of this disease and the absence of standardized diagnostic criteria hinder the identification and treatment of affected individuals (Molnar et al., 2024). Therefore, one approach is to investigate treatments previously used in the pathophysiological processes involved in the development of PCC, such as mitochondrial dysfunction. 

Therapeutic approaches for mitochondrial dysfunction focus on restoring cellular energy production and reducing oxidative stress through antioxidants, such as coenzyme Q10, MitoQ, N-acetylcysteine, alongside lifestyle interventions including tailored exercise and dietary modifications rich in omega-3s and B vitamins (Akanchise and Angelova, 2023; Pavlidou et al., 2024; Molnar et al., 2024). Although improving mitochondrial health shows promise in alleviating some persistent symptoms experienced by post COVID-19 condition patients, significant research limitations remain, including the lack of large-scale clinical trials to validate its efficacy and limited understanding of persistent mechanisms like viral-induced structural damage (Chen et al., 2023a; Michalak et al., 2025). Therefore, identifying mtDNA biomarkers that may indicate mitochondrial dysfunction could help tailor therapeutic approaches to each patient’s needs, thereby improving outcomes.

Mitochondria are essential cytoplasmic organelles involved in key cellular processes, such as energy production, cell death, calcium homeostasis, lipid metabolism, and metabolic signaling. They generate approximately 90% of cellular ATP, primarily through OXPHOS and the TCA, emphasizing the need for these organelles to be studied in depth. Therefore, understanding the role of mitochondria in syndromes such as post COVID-19 condition is crucial for uncovering their causes and consequences, as well as for advancing the development of more effective treatments and preventive strategies for mitochondrial disorders.

Here, we reviewed the current scientific evidence on the interplay between *SARS-CoV-2* infection and mitochondrial biology, with particular emphasis on the role of mitochondria and mtDNA in orchestrating cellular responses to viral challenge. Despite the inherent limitations of this research, notably the lack of studies directly elucidating the pathophysiology of post COVID-19 condition, the synthesis of available data allows us to suggest that mitochondrial dysfunction may be a critical determinant of disease progression, with significant implications for therapeutic strategies.

In this context, mitochondrial pathways, including those related to bioenergetics, redox homeostasis, innate immune signaling, and mtDNA release, may modulate both acute disease severity and the establishment of long-term sequelae. Therefore, we suggest that alterations in mitochondrial function and the mtDNA may act as a convergent axis linking genetic susceptibility, dysregulated immune responses, and chronic inflammation, thereby influencing the progression of COVID-19 and its persistence beyond the acute phase. Such mechanisms contribute to the sustained symptomatology observed in post COVID-19 condition, including fatigue and cardiovascular, muscular, and neurological impairments. Thus, future longitudinal studies with larger cohorts and statistical robustness are needed to elucidate the mechanisms of mitochondrial interactions with the virus and the consequences in the post COVID-19 condition.

## Data Availability

This study did not generate any new data.
